# Agomelatine Augmentation of Escitalopram Therapy in Treatment-Resistant Obsessive-Compulsive Disorder: A Case Report

**DOI:** 10.1155/2012/642752

**Published:** 2012-10-09

**Authors:** Domenico De Berardis, Nicola Serroni, Stefano Marini, Giovanni Martinotti, Francesca Ferri, Gaetano Callista, Raffaella La Rovere, Francesco Saverio Moschetta, Massimo Di Giannantonio

**Affiliations:** ^1^NHS, Department of Mental Health, Psychiatric Service of Diagnosis and Treatment, Hospital “G. Mazzini”, ASL 4, 64100 Teramo, Italy; ^2^Department of Neurosciences and Imaging, Chair of Psychiatry, University “G. D'Annunzio”, 66100 Chieti, Italy; ^3^NHS, Department of Mental Health, Psychiatric Service of Diagnosis and Treatment, Hospital “Maria SS. dello Splendore”, ASL 4, 64021 Giulianova, Italy

## Abstract

Obsessive-compulsive disorder (OCD) is a chronic condition characterized by obsessions or compulsions that cause distress or interfere with functioning. Selective serotonin reuptake inhibitors are the first-line strategy in the treatment of OCD, but approximately 40% to 60% of patients with OCD fail to respond to them. Several augmentation strategies have been proposed, including the use of atypical antipsychotics and antidepressant combinations. In the present paper we describe the case of a young female patient suffering from severe treatment-resistant OCD who remitted as a result of agomelatine augmentation of escitalopram therapy.

## 1. Introduction

Obsessive-compulsive disorder (OCD) is a chronic and disabling anxiety disorder with a fluctuating course, characterized by obsessions or compulsions that cause severe distress and interfere with patient's functioning [1]. Selective serotonin reuptake inhibitors (SSRIs) are the first-line strategy in the treatment of OCD, but approximately 40% to 60% of patients with OCD fail to respond to them [2]. 

Agomelatine, a naphthalenic compound chemically designated as N-[2-(7-methoxynaphth-1-yl)ethyl] acetamide or S-20098, is a newly developed antidepressant drug with selective agonism for melatonergic MT1/MT2 receptors and antagonism for 5-HT2c receptors [3]. It has been demonstrated that agomelatine may increase dopamine and noradrenaline levels in the frontal cortex, also stimulating cell proliferation and neurogenesis [4, 5]. Efficacy of agomelatine in the treatment of major depression has been demonstrated in several studies [6, 7]. 

It has been reported that agomelatine may be beneficial in the treatment of OCD, although data are limited mainly to case series [8, 9], with initial observations of some efficacy in treatment resistance [10]. 

The case presented below describes the case of a young female patient suffering from severe treatment resistant OCD who was successfully treated with agomelatine augmentation of escitalopram therapy.

## 2. Case Report

A 25-year-old female white collar came to our observation at the outpatient facility of the Psychiatric Service of Diagnosis and Treatment, after a consultation at the local emergency room due to a vulvar pruritus and dermatitis as a consequence of compulsive washing. She was not engaged and lived alone, not reporting any stressful situations related to the working or marital status. She did not report a conflictual relationship with her parents and older sister and said that “they always help me to face up my problems.” She had not been misusing alcohol or illicit drugs and did not smoke. Her history dated to almost five years previously when she insidiously started to experience obsessive-compulsive symptoms (mainly the fear of contamination from touching various things she considered dirty). She was diagnosed with OCD at the age of 21, during her first visit to a private psychiatrist. 

At time of our first consultation, her symptoms included ritual washing of hands and genitals in response to contamination obsessions as well as checking compulsions. These symptoms were severe, as evidenced by the fact that she was diagnosed as having a severe dermatitis at both hands and genitals. These obsessive-compulsive behaviors caused her to spend considerable time and energy that severely interfered with her daily activities and working status. Moreover, she reported difficulty of sleeping as a consequence to the need to perform washing during night at scheduled times. She reported no personal or family history of neither neurological nor mental disorders, other than OCD. Insight was present, and the patient said that “…these thoughts and behaviors are absurd, but I cannot resist as anxiety keeps to growing up. I feel calm after washing my hands and genitals, but this is ridiculous and silly!”

Prior to our clinical assessment, she had been treated by several private psychiatrists first with fluvoxamine up to 300 mg/day for approximately one year, then with sertraline 200 mg/day and alprazolam 1.5 mg/day for approximately 10 months. Quetiapine up to 450 mg/day was added for almost six months to sertraline but was discontinued due to adverse effects. A subsequent trial with clomipramine 150 mg/day was stopped after three months due to drowsiness and severe constipation, without observed improvement.

She reported several treatment-related side effects, including weight gain (mainly with fluvoxamine and sertraline/quetiapine combination), sexual dysfunctions (mainly with fluvoxamine and sertraline), severe sedation (sertraline/quetiapine combination), and a relative lack of efficacy (all drugs). Furthermore, throughout the first six months of fluvoxamine treatment, she received concomitant cognitive-behavioral psychotherapy but reported no beneficial effects. At the time of our first interview, she was taking escitalopram 30 mg/day at the morning for approximately 5 months without improvement but without reported severe adverse effects except mild appetite increase with moderate weight gain. 

Based on the structured clinical interview for DSM-IV, she was diagnosed with obsessive-compulsive disorder and scored 33 on the Yale-Brown Obsessive-Compulsive Scale (Y-BOCS). Insight was present (score of 0 on item number 11 of the Y-BOCS). Also, she displayed only mild depressive symptoms (as evidenced by a Montgomery and Asberg Rating Scale score of 16), and they were mainly due to secondary demoralization. Before starting agomelatine' augmentation, a comprehensive physical examination and laboratory testing were carried out. Laboratory results, brain CT, electroencephalogram, electrocardiogram, and chest radiograph did not show any problem. Also, substance and alcohol use were ruled out on the basis of history taking and laboratory screening. Moreover, she had no evidence of current or past depressive or manic episodes or psychotic symptoms.

Agomelatine 25 mg/day at bedtime was prescribed in addition to escitalopram. She refused psychotherapeutic intervention, owing mainly to “my financial status” and a perceived lack of efficacy.

After three weeks of treatment with agomelatine 25 mg/day, we observed a little reduction in OCD symptoms (Y-BOCS score of 25), while MADRS score dropped to 6. Agomelatine/escitalopram therapy did not cause any adverse effects, and the patient stated she was willing to continue to be treated with this combination as she noticed a significant improvement in sleep quality and a subjective small symptom relief. It was proposed to the patient to increase the agomelatine dosage, but she refused because she was feeling better, trusting in further improvement with the same dosage. By the end of the fourth week, a moderate decline in symptomatology was observed (Y-BOCS score of 20). Three weeks later, the patient reported a further gradual subjective and objective improvement (Y-BOCS score of 13). After other five weeks of constant improvement, the patient fully remitted and obtained a score of 6 on the Y-BOCS. No adverse effects were observed during this period. During the following visits, taking place every two or three weeks, the full clinical remission was maintained, and escitalopram gradually tapered to 20 mg/day without symptoms' worsening. 

At the time of the last examination (June 2012), the patient was taking agomelatine 25 mg/day and escitalopram 20 mg/day with no OCD symptoms (Y-BOCS score of 5) and no adverse effects. The patient provided informed consent to present this paper. 

## 3. Discussion

In the present paper, agomelatine augmentation of escitalopram remarkably improved OCD symptomatology. It is well documented that, compared to depression, higher doses of SSRIs are usually required to treat OCD [11]. Several studies have reported that OCD patients who are nonresponders to usual therapeutic antidepressant doses might respond to much higher doses [12]. In line with these evidences, in a previous case report, we found that higher doses of agomelatine (50 mg/day) leaded to therapeutic response with full clinical remission (defined as a Y-BOCS score <7) [9]. However, in the present case, agomelatine dose was 25 mg/day suggesting a potential efficacy of such augmentation and not an effect of agomelatine per se. This finding suggests that escitalopram augmentation with lower doses of agomelatine might represent a valid treatment option in OCD, especially when, as observed in the present case, the obsessive-compulsive symptoms are severe and cause significant functional disability. Moreover, it is unlikely that treatment response may be the result of a pharmacokinetic interaction as agomelatine is mainly metabolized by CYP1A2 [3] whereas demethylation of escitalopram involves the cytochrome P450 CYP2C19, CYP2D6, and CYP3A4 isozymes [13].

Nevertheless, it is possible to hypothesize that agomelatine's tolerability and safety profile especially when given in association to escitalopram may counteract potential adverse effects of such drugs (such as weight gain) that may contribute to therapeutic effect [3]. 

It is possible to hypothesize that agomelatine's antiobsessive properties may be due to its combined action on both the melatonergic MT1/MT2 and the 5-HT2c receptors in the frontal cortex [2]. In fact, it has been demonstrated that agomelatine may increase dopamine and noradrenaline levels in the frontal cortex, mainly through the blockade of 5-HT2c receptors' inhibitory input to cortical dopaminergic and adrenergic pathways [14]. The latter mechanism of action, combined with the restored serotonin transmission due to escitalopram, may explain the positive outcome observed in this case. 

Moreover, the positive effect of agomelatine augmentation may be due to its efficacy in normalizing disturbances of circadian rhythms [15]. In fact, the observed improvement in the patient's anxiety-related sleep disturbances following agomelatine introduction may further play a role in improvement of psychiatric' status. It has been demonstrated that patients with OCD may show hypervigilance and problems falling asleep [16, 17]. Furthermore, Monteleone et al. [18] reported, in OCD patients, an hyperactivity of the hypothalamic-pituitary-adrenal axis, with an increased secretion of adrenocorticotropic hormone and cortisol and a reduced secretion of melatonin. On the basis of such observations, it is also possible to hypothesize that the restoration of circadian rhythms, such as the improvement of sleep efficiency together with the disappearance of intrasleep awakening, through MT1 and MT2 agonism due to agomelatine augmentation, may have contributed to improve symptomatology confirming the observation of da Rocha and Correa [10].

## 4. Conclusion

The present paper supports the notion that agomelatine augmentation of escitalopram may be effective in the treatment of OCD. This strengthens the hypothesis that agomelatine may be effective in treating anxiety disorders [8, 19, 20]. However, although encouraging, this is only a paper and therefore, prospective double-blind, placebo-controlled studies on larger samples are undoubtedly needed. 
